# Supplementary material for: Unusual topological polar texture in moiré ferroelectrics
Source: Nat Commun. 2025 Jul 1;16:5451. doi: 10.1038/s41467-025-60647-y (PMC12217333; doi:10.1038/s41467-025-60647-y)
Supplement: Supplementary file 1 — Supplementary Information [file 41467_2025_60647_MOESM1_ESM.pdf]

Supplementary Information:

Unusual topological polar texture in moiré ferroelectrics

Yuhao Li[1,2], Yuanhao Wei[3], Ruiping Guo[4,5], Yifei Wang[4], Hanhao Zhang[1], Takashi Taniguchi[6], Kenji Watanabe[7], Yan Shi[3], Yi Shi[1,*], Chong Wang[4,*], Zaiyao Fei[1,2,*]

1. National Laboratory of Solid-State Microstructures, School of Electronic Science and Engineering and Collaborative Innovation Center of Advanced Microstructures, Nanjing University, Nanjing 210093, Jiangsu, China
2. National Key Laboratory of Spintronics, Nanjing University, Suzhou 215163, Jiangsu, China
3. State Key Laboratory of Mechanics and Control for Aerospace Structures, Nanjing University of Aeronautics and Astronautics, Nanjing 210016, Jiangsu, China
4. State Key Laboratory of Low Dimensional Quantum Physics and Department of Physics, Tsinghua University, Beijing 100084, China
5. Institute for Advanced Study, Tsinghua University, Beijing 100084, China
6. Research Center for Materials Nanoarchitectonics, National Institute for Materials Science, 1-1 Namiki, Tsukuba 305-0044, Japan
7. Research Center for Electronic and Optical Materials, National Institute for Materials Science, 1-1 Namiki, Tsukuba 305-0044, Japan

[*]Correspondence to: zyfei@nju.edu.cn, chongwang@mail.tsinghua.edu.cn and yshi@nju.edu.cn

Supplementary Notes:

Supplementary Note 1. Removing background signals in VPFM measurement 2

Supplementary Note 2. Buckling effect in VPFM measurements 2

References: 24

Supplementary Note 1. Removing background signals in VPFM measurement

In analogous to removing background signals in LPFM images of moiré superlattices[1,2] we also apply the vectorial decomposition method[3] to VPFM images, as schematically illustrated in Fig. S4e. Specifically, the background VPFM signal ($\mathbf{d_b}$) of each scan line is obtained by averaging the measured averaging VPFM signal of AB ($\bar{\mathbf{d}}_{\mathbf{AB}} = \sum_{j=1}^{n}(\mathbf{d}^{\mathbf{j}}_{\mathbf{m,AB}})/n$) and BA ($\bar{\mathbf{d}}_{\mathbf{BA}} = \sum_{j=1}^{n}(\mathbf{d}^{\mathbf{j}}_{\mathbf{m,BA}})/n$) domains $\mathbf{d_b} = A_\mathrm{b}\mathrm{e}^{i\theta_\mathrm{b}} = (\bar{\mathbf{d}}_{\mathbf{AB}} + \bar{\mathbf{d}}_{\mathbf{BA}})/2$, where $\mathbf{d}^{\mathbf{j}}_{\mathbf{m,AB}} = A^{\mathrm{j}}_{\mathrm{m,AB}}\mathrm{e}^{i\theta^{\mathrm{j}}_{\mathrm{m,AB}}}$ and $\mathbf{d}^{\mathbf{j}}_{\mathbf{m,BA}} = A^{\mathrm{j}}_{\mathrm{m,BA}}\mathrm{e}^{\mathrm{i}\theta^{\mathrm{j}}_{\mathrm{m,BA}}}$, $A^{\mathrm{j}}_{\mathrm{m,AB}}$ and $\theta^{\mathrm{j}}_{\mathrm{m,AB}}$ are measured amplitude and phase at the *j*-th point inside AB domain, $A^{\mathrm{j}}_{\mathrm{m,BA}}$ and $\theta^{\mathrm{j}}_{\mathrm{m,BA}}$ are measured amplitude and phase at the *j*-th point inside BA domain, *n* is the number of points for AB or BA domain. To distinguish AB and BA domains, two masks are required to label the identity of every point inside domains. The decoupled VPFM signal can be expressed by $\mathbf{d}^{\mathbf{j}} = \mathbf{d}^{\mathbf{j}}_{\mathbf{m}} - \mathbf{d_b}$.

Supplementary Note 2. Buckling effect in VPFM measurements

Ideally, LPFM captures the in-plane deformation of a sample in response to an applied electric field, whereas VPFM captures the out-of-plane deformation. In experiment, the measured LPFM signal (S_{LPFM}) records the torsion of the cantilever. Thus, only the perpendicular component of in-plane polarization ($P_{\perp} = P_{\mathrm{xy}}\cdot\sin(\varphi)$) would contribute to it, as shown in Fig. S9b, where φ is the angle between cantilever and polarization. The measured VPFM signal, on the other hand, usually records the bending of cantilever (S_{Bending}) as a result of the out-of-plane polarization (P_z), as shown in Fig. S9d. However, the parallel component of in-plane polarization ($P_{//} = P_{\mathrm{xy}}\cdot\cos(\varphi)$) can result in the buckling of cantilever which also contributes to the VPFM signal (S_{Buckling}), as illustrated in Fig. S9c. Therefore, the LPFM and VPFM signals can be expressed in the following[4,5]:

$$S_{\mathrm{LPFM}} = S_{\mathrm{Torsion}} = \alpha\cdot d_{31}\cdot P_{\perp} = \alpha\cdot d_{31}\cdot P_{\mathrm{xy}}\cdot\sin(\varphi) \quad (1)$$

$$S_{\mathrm{VPFM}} = S_{\mathrm{Bending}} + S_{\mathrm{Buckling}} = \beta\cdot d_{33}\cdot P_\mathrm{z} + \gamma\cdot d_{31}\cdot P_{//} = \beta\cdot d_{33}\cdot P_\mathrm{z} + \gamma\cdot d_{31}\cdot P_{\mathrm{xy}}\cdot\cos(\varphi) \quad (2)$$

where α, β, and γ are stiffness coefficients, d_{33} and d_{31} are out-of-plane and in-plane piezoelectric

coefficients.

Except for aligning DWs perpendicular to the cantilever, the buckling effect can also be suppressed by optimizing the laser position[6]. Figure S13 presents decoupled VPFM images of thBN4 with weak buckling effect for all DWs simultaneously.

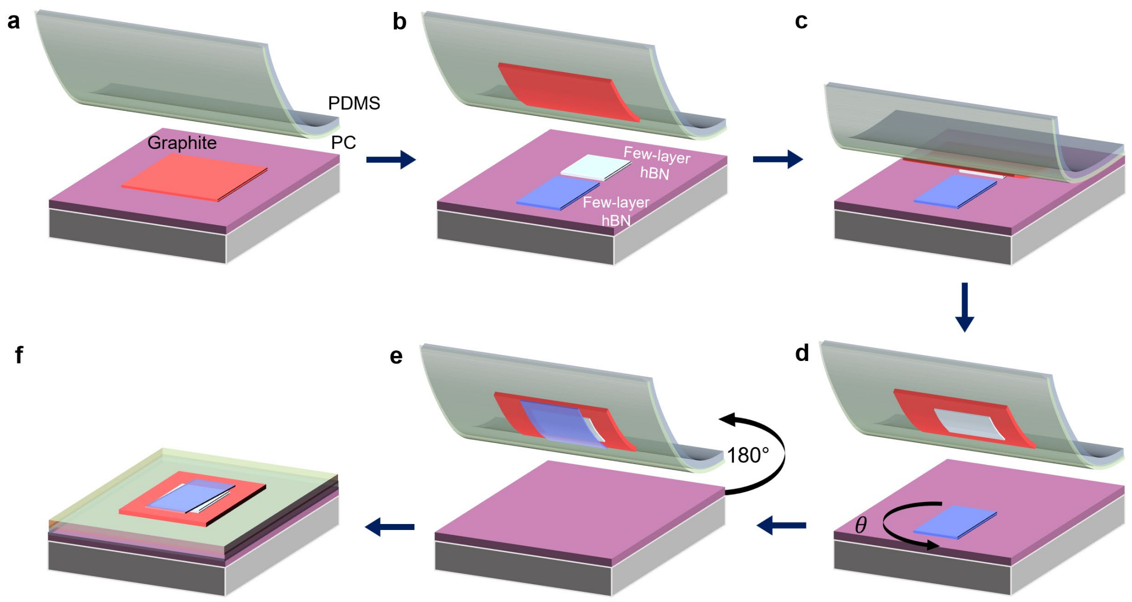

Fig. S1 Schematic illustration of the sample fabrication process flow. A PC/PDMS stamp was used to pick up a graphite flak (**a**), then half of a few-layer hBN (**b, c**), followed by the second half of the few-layer hBN (**d**) with a desired twist angle. To access the surface of twisted hBN, the PC film was released from stamp by a thermal release tape (**e**), and then flipped over and placed on a Si/SiO_2 chip (**f**).

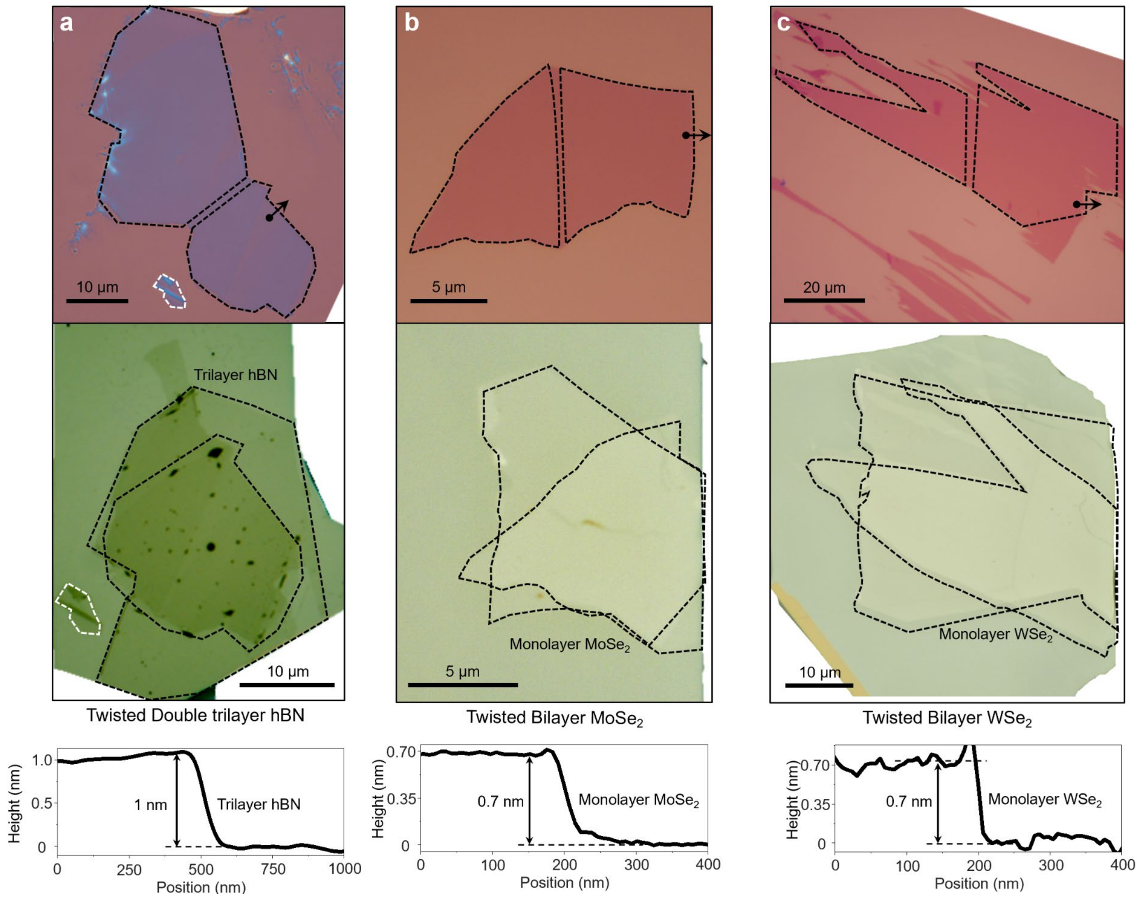

Fig. S2 Representative optical images and corresponding topographies of twisted double trilayer hBN (**a**), twisted bilayer $MoSe_2$ (**b**) and twisted bilayer WSe_2 (**c**) samples. The linecuts in the bottom insets shows the thickness of the constituent flakes.

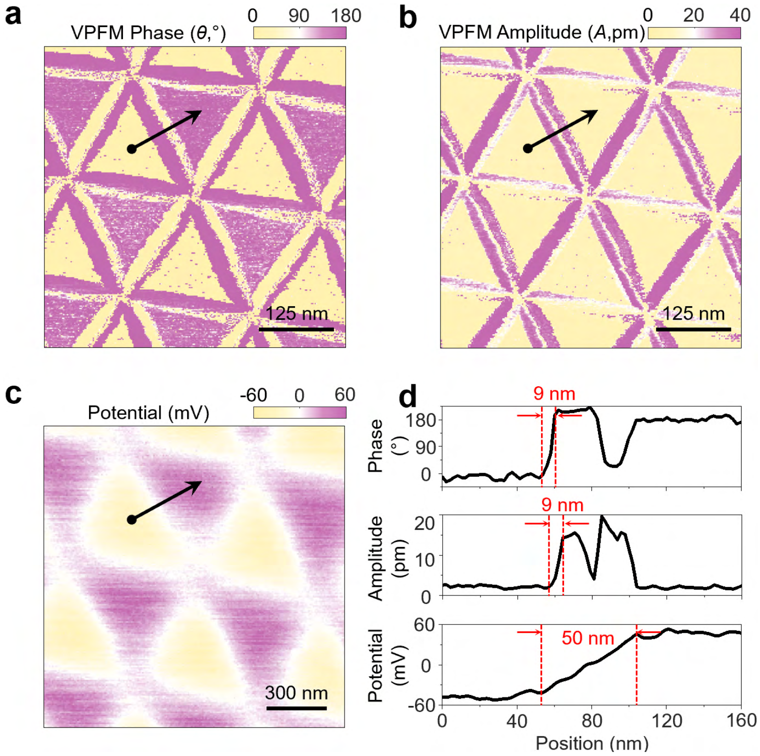

Fig. S3 In-situ VPFM (a, b) and KPFM (c) measurements on thBN2. The linecuts in (**d**) demonstrate spatial resolutions of 9 nm (VPFM) and 50 nm (KPFM), determined from the transition across the same sharp step. The twist angle of this region is around 0.065°.

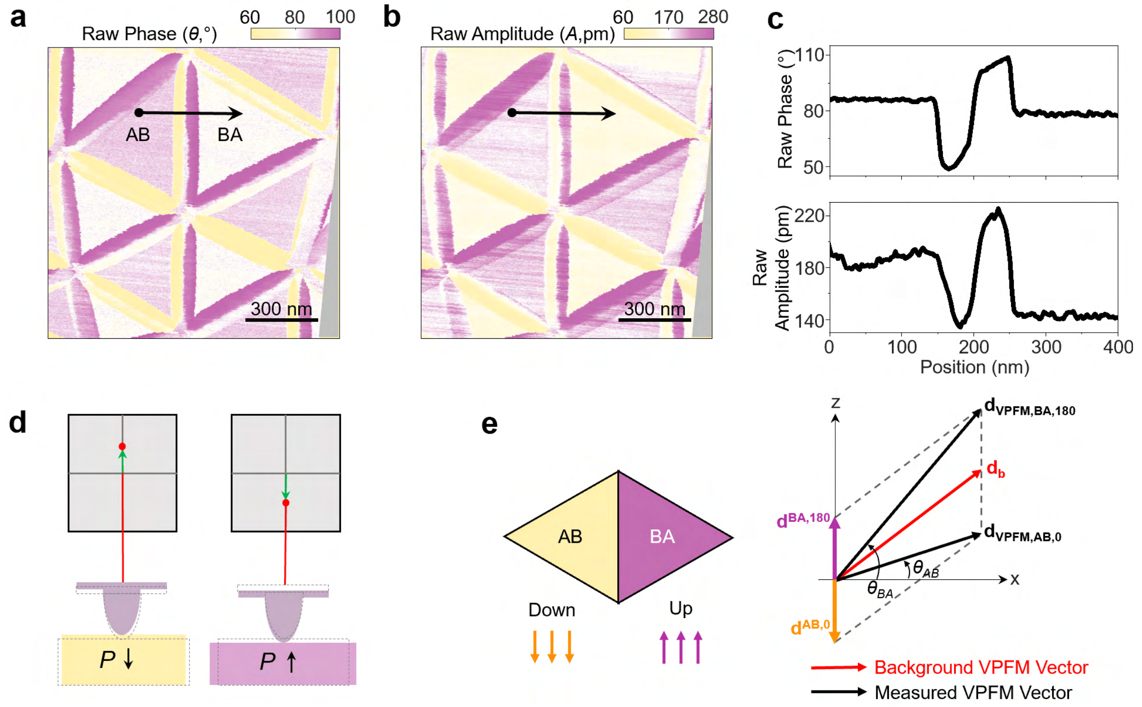

Fig. S4 VPFM background signal decoupling processes. a-c Raw VPFM images and linecuts of the thBN1 in Fig. 1d, e. The raw phase difference between AB and BA domains is only 6°, and the amplitude change between AB and BA is over 46 pm. These are inconsistent with the expected 180° phase change and identical amplitude between AB and BA domains. **d** Working principle of VPFM. **e** Principle of vectorial decomposition for VPFM signal.

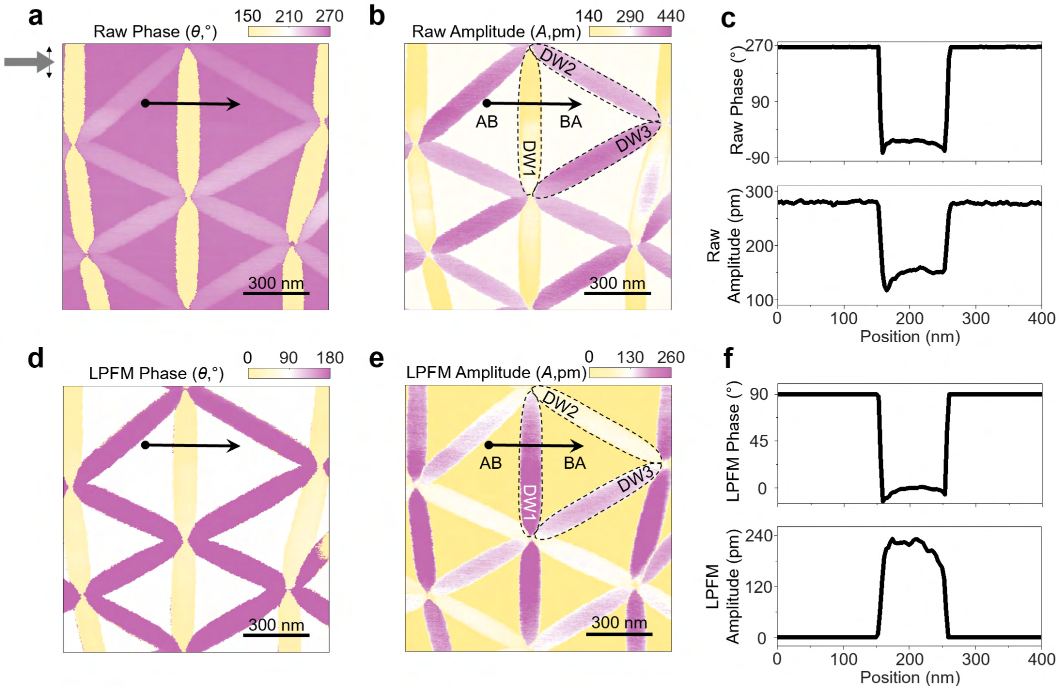

Fig. S5 Raw (a-c) and decoupled (d-f) LPFM images and linecuts of thBN1 in Fig. 2a, showing the effect of background signal decoupling.

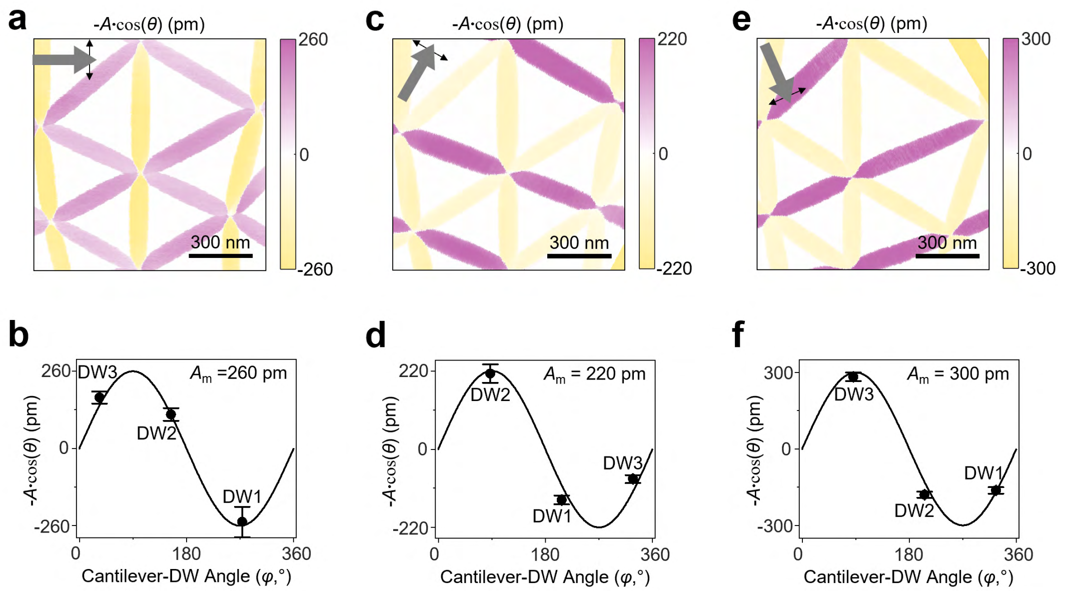

Fig. S6 Detailed analysis of the angle-dependent LPFM results in Fig. 2d. a,c,e In-phase LPFM signal (-A•cos(θ)) images corresponding to the phase and amplitude images in Fig. 2a-c for $\psi = 0$, 60º, and 300º. **b,d,f** Corresponding sine relation between in-phase LPFM signal and cantilever-DW angle (φ) in **a,c,e**. The amplitudes (A_m) of three sine fits vary from 220 to 300 pm due to variations of quantity factors (Q) for different scans. To compare different scans, we normalized the amplitude as in Fig. 2 of the main text, where $A_n = A/A_m$.

Reconstructed In-plane Polarization ($\mathbf{P}_{xy}$)

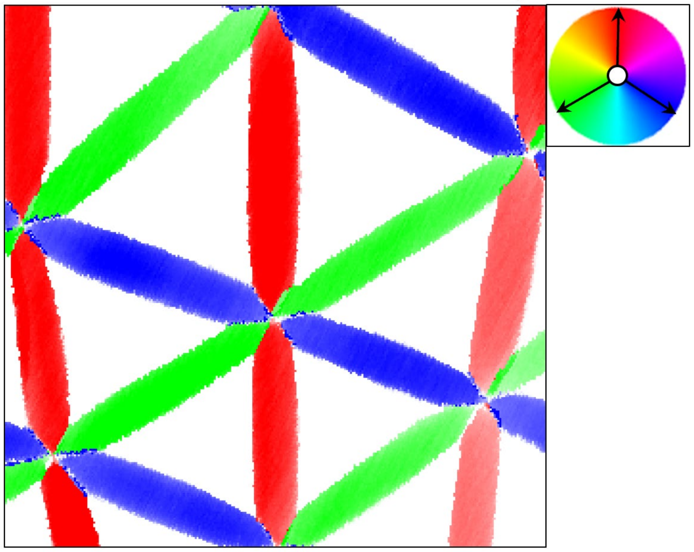

Fig. S7 Reconstructed in-plane polarization of thBN1 with data from Fig.2 a-c.

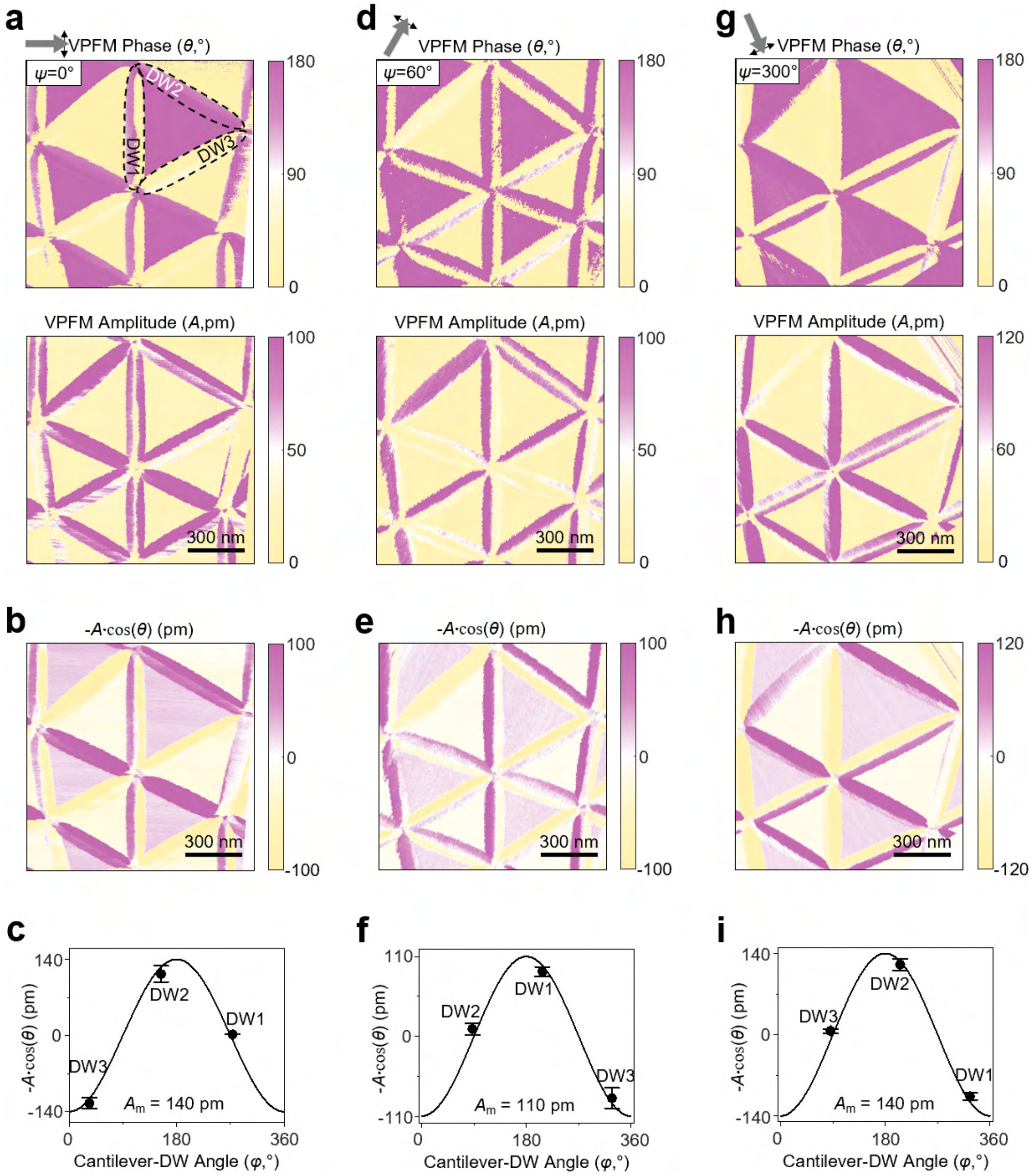

Fig. S8 Detailed analysis of the angle-dependent VPFM results in Fig. 2d. a,d,g Decoupled VPFM images of the same sample area of thBN1 at three cantilever-sample angles (ψ = 0°, 60°, and 300°). **b,e,h** In-phase VPFM signal (-A•cos(θ)) images. **c,f,i** Corresponding cosine relation between averaged in-phase VPFM signal of DW and cantilever-DW angle (φ) in **b, e** and **h**.

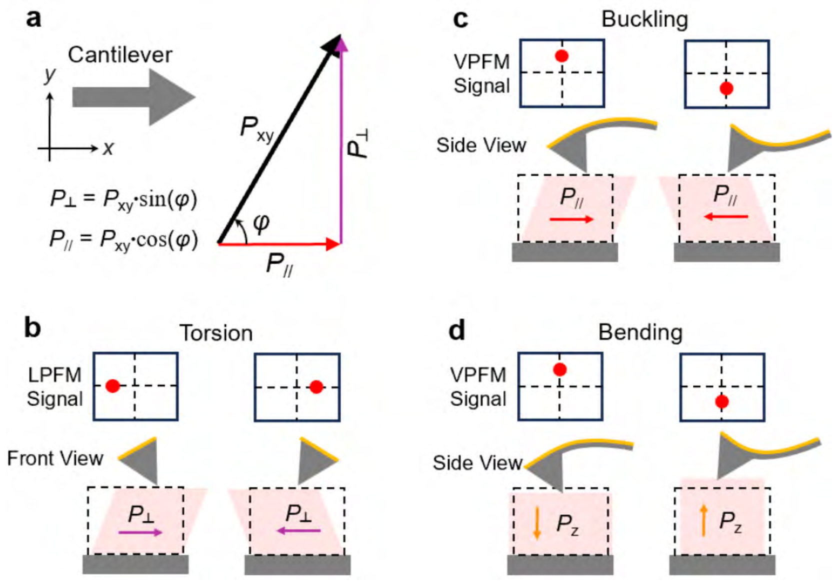

Fig. S9 Cantilever motions in vector PFM measurements. a Illustration of an in-plane polarization decomposition. $P_\perp$ and $P_{//}$ correspond to different components of P_{xy} that are perpendicular and parallel to the cantilever, respectively. **b** Torsional motion in LPFM measurements, where only $P_\perp$ generates LPFM signals. **c** Buckling motion in VPFM measurements due to $P_{//}$ component of P_{xy}. Therefore, the in-plane polarization induced VPFM signal is named as 'buckling effect'. **d** Bending motion in VPFM measurements due to P_z.

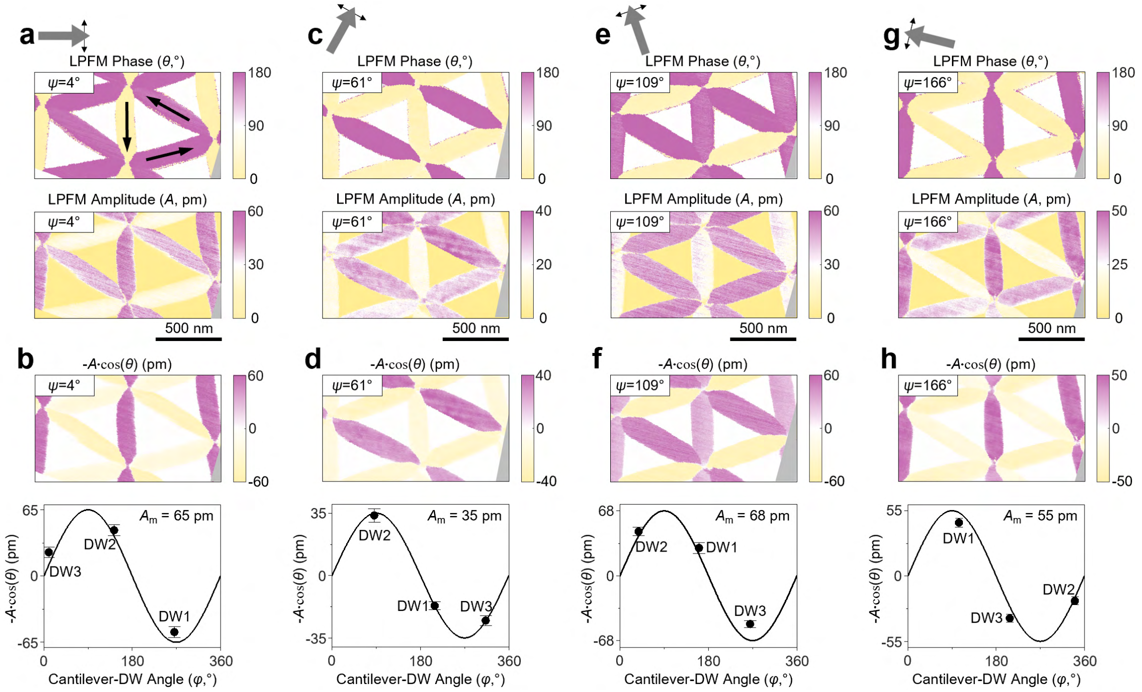

Fig. S10 LPFM results of thBN3 at different sample-cantilever angles. a Schematic of measurement setup and corresponding LPFM images of thBN3 for ψ = 4°. **b** In-phase LPFM signal (-A·cos(θ)) images and corresponding sinusoidal relation between in-phase LPFM signal of DW and cantilever-DW angle (φ) in **a**. **c-h** LPFM results for ψ varying from 61° to 166°. **w** Summarized results and cosinoidal fit between normalized in-phase VPFM signal (-A_n·cos(θ)) and cantilever-DW angle (φ), where A_n=A/A_m, A_m is the amplitude of sine fit, which indicates the maximum LPFM amplitude.

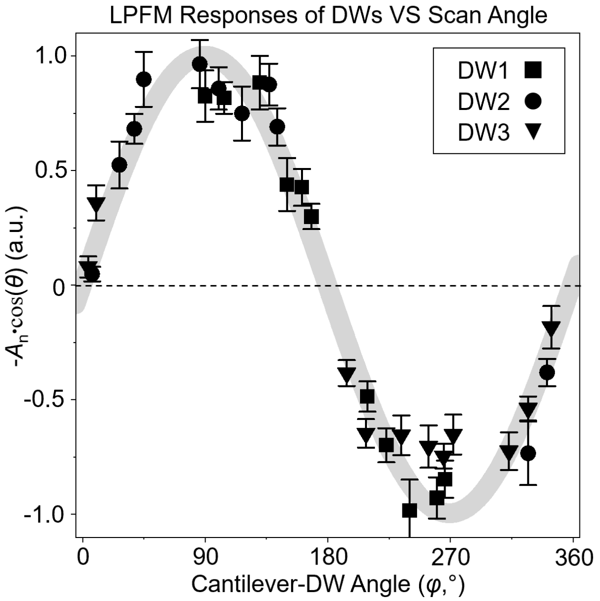

Fig. S11 Summarized LPFM results of Fig. S10 and sine fit between normalized in-phase LPFM signal ($-A_n \cdot \cos(\theta)$) and cantilever-DW angle (φ), where $A_n = A/A_m$, A_m is the amplitude of sine fit, which indicates the maximum LPFM amplitude.

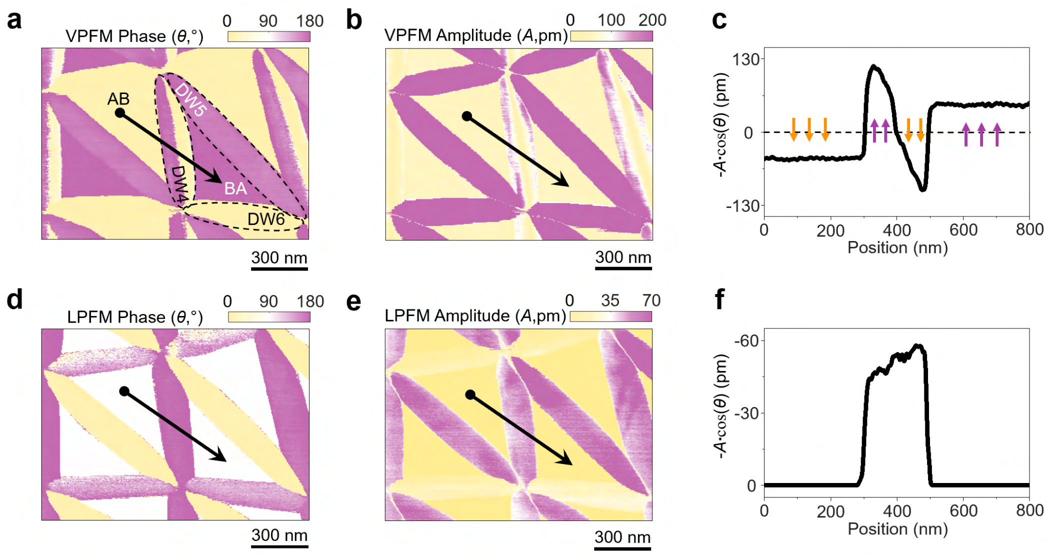

Fig. S12 VPFM and LPFM results of thBN3 for $\psi = 0°$. a,b Decoupled VPFM phase and amplitude images. DW4 shows clear 180° phase change. **c** Linecut of VPFM signal ($-A\cdot\cos(\theta)$) along the black lines in **a** and **b**. **d,e** LPFM images of the same area in **a**. **f** Linecut of LPFM signal ($-A\cdot\cos(\theta)$) along the black lines in **d** and **e**.

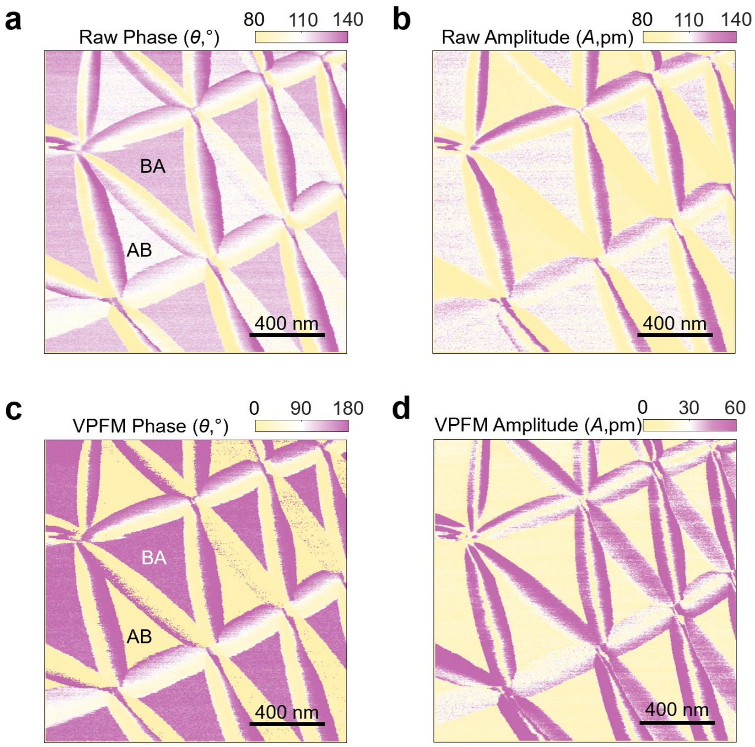

Fig. S13 VPFM results of thBN4 with negligible buckling effect. Raw (**a,b**) and decoupled (**c,d**) VPFM images of thBN4.

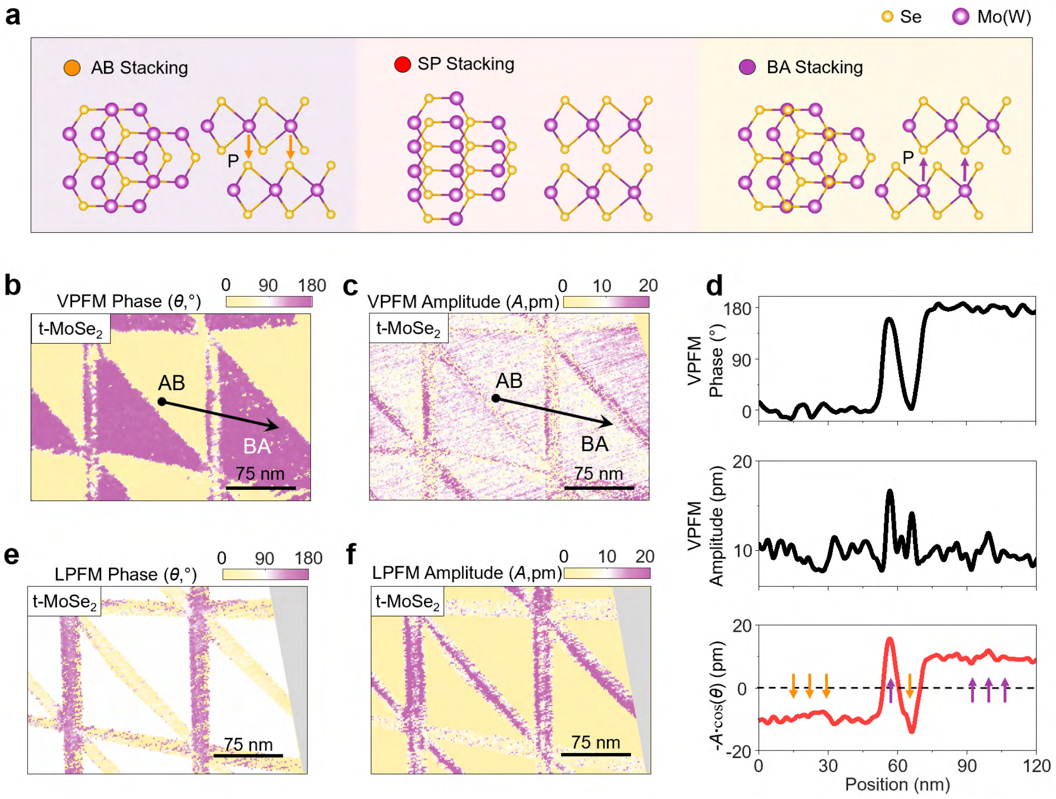

Fig. S14 VPFM and LPFM measurements on a twisted bilayer $MoSe_2$ sample. a Schematic of AB, BA and SP stacking domains of a twisted TMDC moiré superlattice. **b,c** VPFM phase and amplitude images of a twisted bilayer $MoSe_2$ sample. **d** Phase, amplitude and in-phase VPFM signal along the black arrowed lines in **b** and **c**. **e,f** In-situ LPFM phase and amplitude images of t-$MoSe_2$ in **b** and **c**.

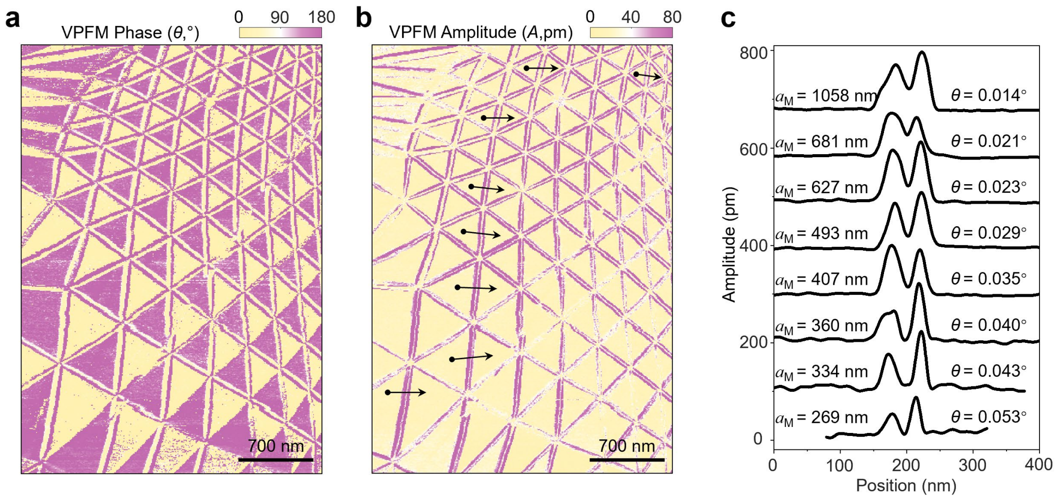

Fig. S15 VPFM measurements of thBN5 with nonuniform twist angles. a,b VPFM phase and amplitude images. **c** Linecuts of amplitude across nearly vertical AB/BA domain walls in **b**. The amplitude signals are offset for clarity. No significant changes are observed as the moiré periodicity (a_M) increases from 269 nm (θ_T = 0.053°) to 1058 nm (θ_T = 0.014°).

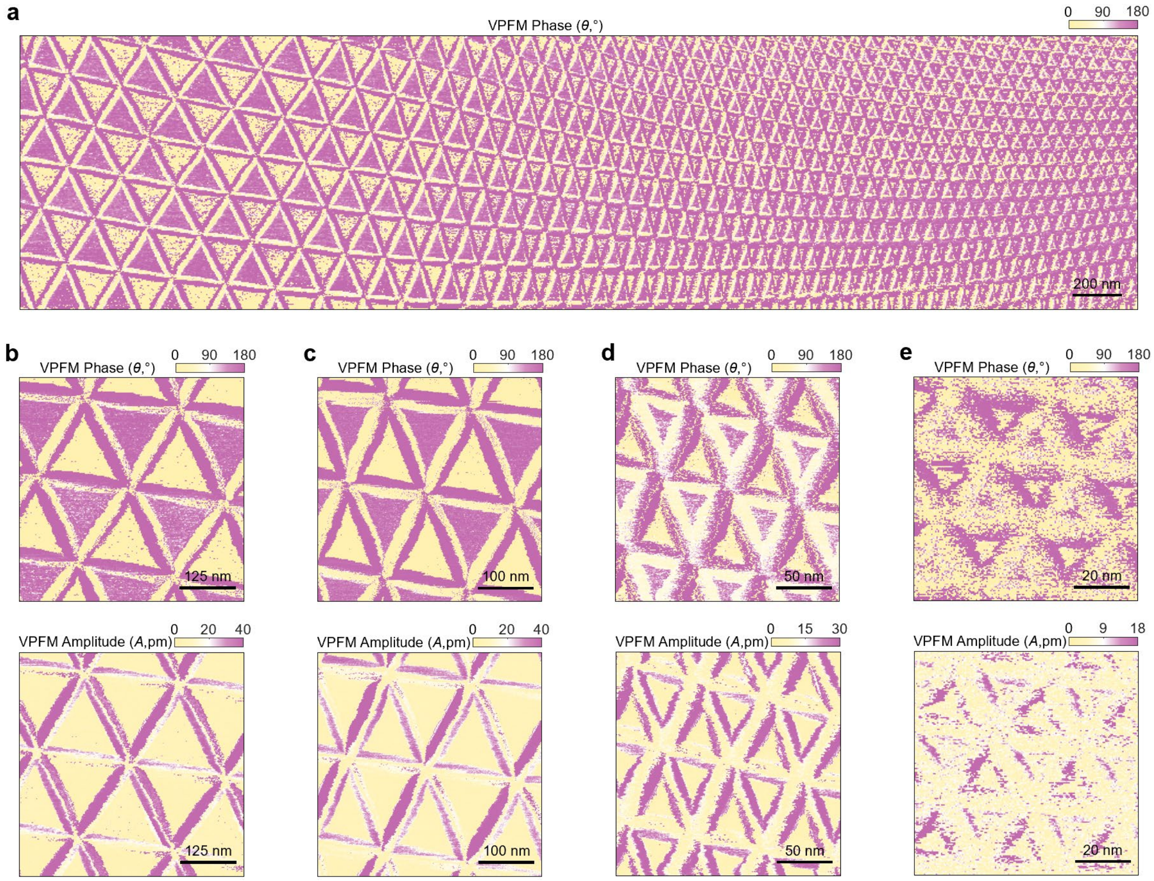

Fig. S16 VPFM measurements of thBN2 with nonuniform twist angles. a VPFM phase image of large scan. **b-e** Zoom-in scans of the selected regions with twist angles vary from 0.064° to 0.375°. The observed multiple sign reversal is robust over the twist angle range of 0.064° to 0.375°. VPFM scans on this sample also show a weak cantilever buckling effect as Fig. S13.

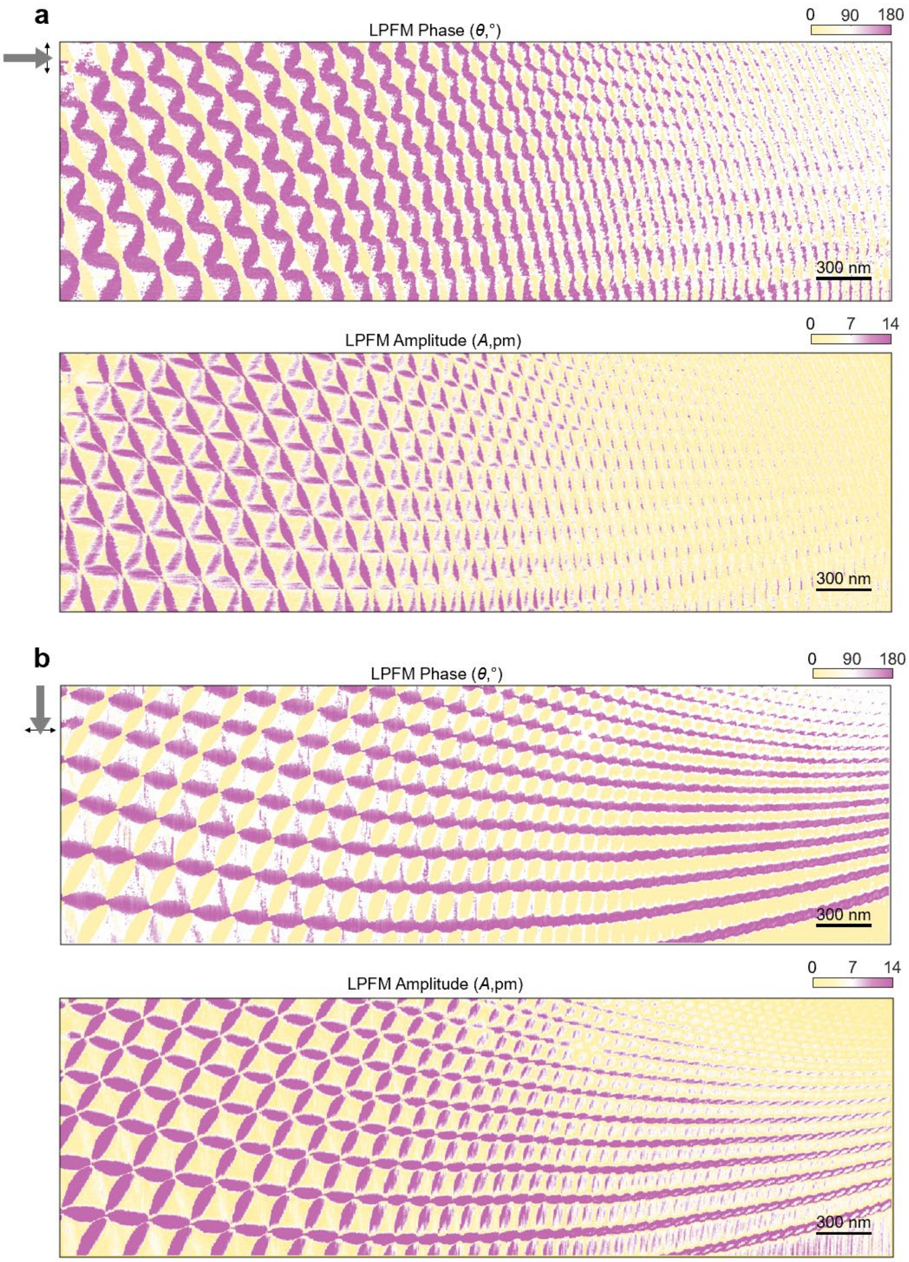

Fig. S17 In-situ LPFM images of the same sample as in Fig. S16 with sample-cantilever angles of 0° (a) and 90° (b), respectively. The twist angles of twisted hBN sample vary from 0.064° to 0.375°.

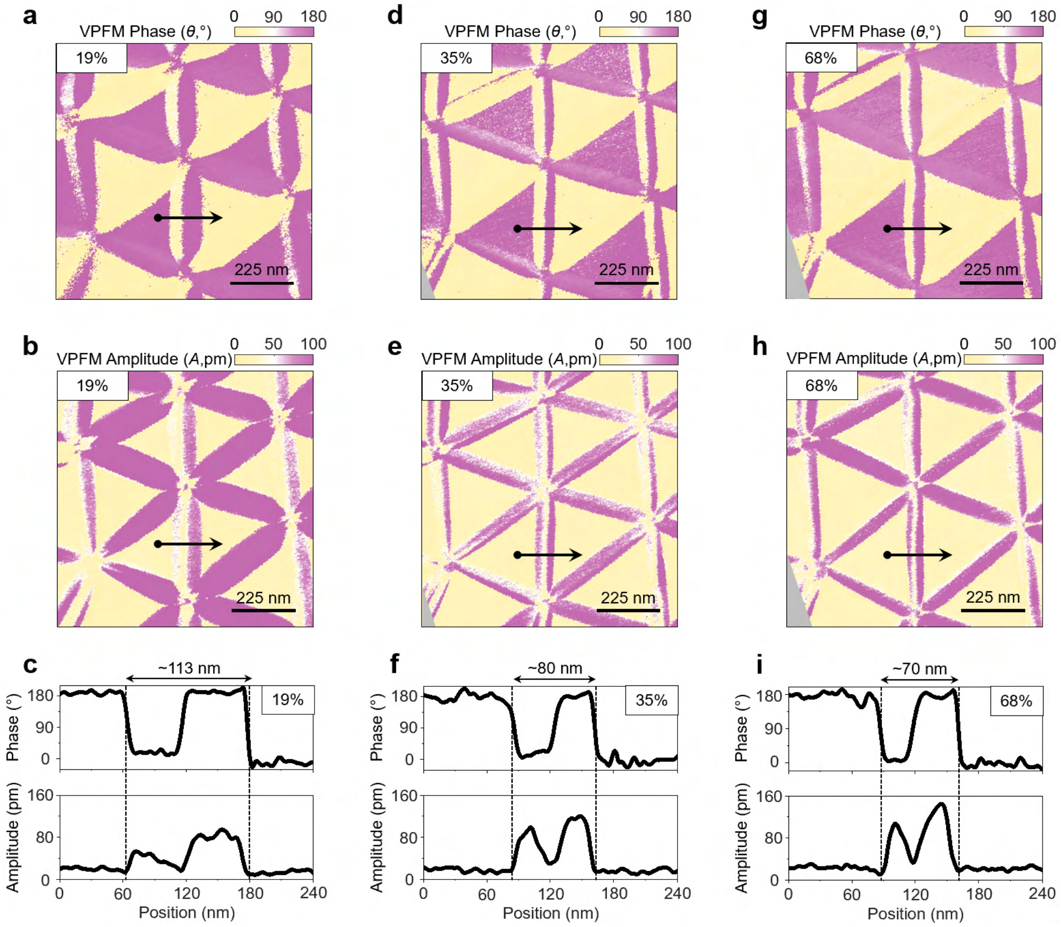

Fig. S18 VPFM measurements of thBN2 under different relative humidity (19%, 35% and 68%). a-i VPFM phase images, amplitude images and corresponding linecuts under 19% (**a-c**), 35% (**d-f**) and 68% (**g-i**) RH, respectively. The spatial resolution increases with relative humidity in this range. The twist angle of this region is around 0.035°.

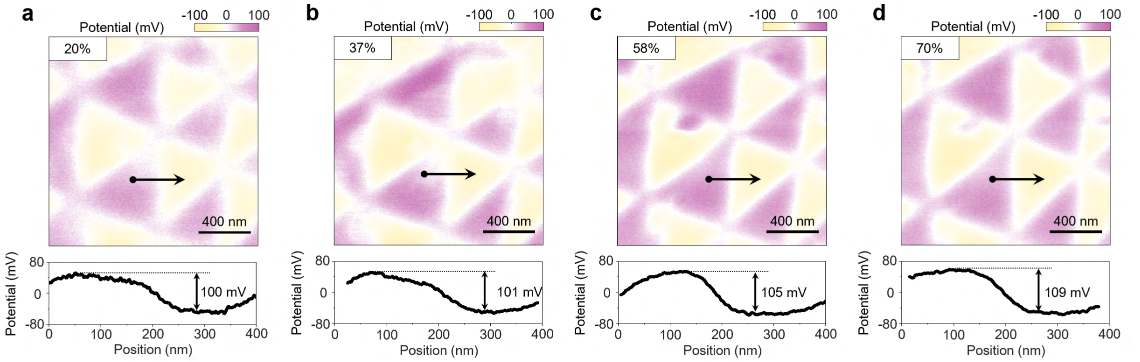

Fig. S19 KPFM measurements of thBN2 under different relative humidity (20%, 37%, 58% and 70%). **a-d** Surface potential image and corresponding line cut with 20%, 37%, 58% and 70% RH. The potential difference between AB and BA stackings keeps almost constant (~105 mV). Note the KPFM is performed on a different region of thBN2, where the twist angle is around 0.02°.

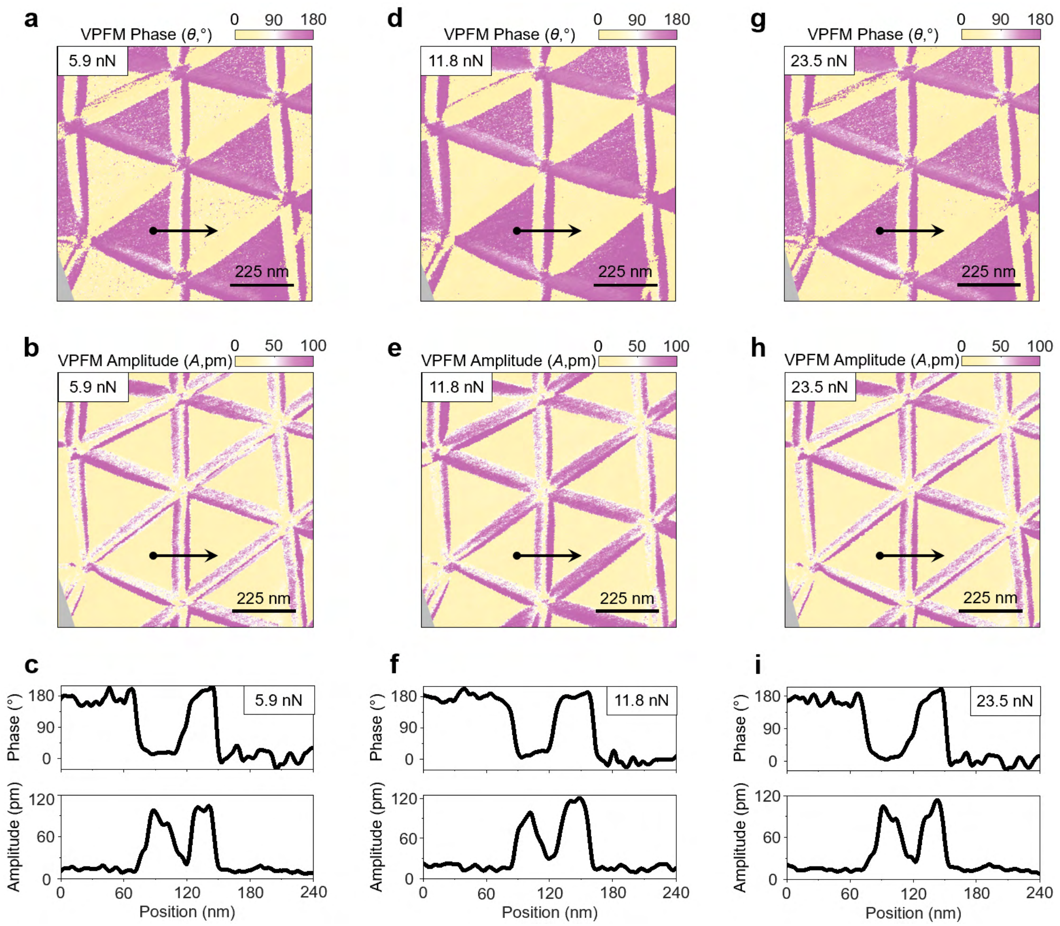

Fig. S20 Force-dependent VPFM measurements of thBN2. a-i VPFM phase images, amplitude images and corresponding linecuts with 5.9 nN (**a-c**), 11.8 nN (**d-f**) and 23.5 nN (**g-i**) normal force, respectively. No significant change is observed for different forces in this range.

References:

1 Li, Y. *et al.* Unraveling Strain Gradient Induced Electromechanical Coupling in Twisted Double Bilayer Graphene Moiré Superlattices. Adv. Mater. 33, 2105879 (2021).

2 Zhang, H. *et al.* Layer-Dependent Electromechanical Response in Twisted Graphene Moiré Superlattices. ACS Nano 18, 17570-17577 (2024).

3 Jungk, T., Hoffmann, Á. & Soergel, E. Quantitative analysis of ferroelectric domain imaging with piezoresponse force microscopy. Appl. Phys. Lett. 89, 163507 (2006).

4 He, L. *et al.* Buckling Effect Induced Abnormal Out of Plane Domain Stripe in BiFeO3 Single-Crystalline Thin Film Investigated by Piezoresponse Force Microscopy. Ferroelectrics 492, 59-68 (2016).

5 Kalinin, S. V., Gruverman, A. & Bonnell, D. A. Quantitative analysis of nanoscale switching in SrBi2Ta2O9 thin filmsby piezoresponse force microscopy. Appl. Phys. Lett. 85, 795-797 (2004).

6 Alikin, D. O. *et al.* In-plane polarization contribution to the vertical piezoresponse force microscopy signal mediated by the cantilever "buckling". Appl. Surf. Sci. 543 (2021).
